# Uncovering a superfamily of nickel-dependent hydroxyacid racemases and epimerases

**DOI:** 10.1038/s41598-020-74802-6

**Published:** 2020-10-22

**Authors:** Benoît Desguin, Julian Urdiain-Arraiza, Matthieu Da Costa, Matthias Fellner, Jian Hu, Robert P. Hausinger, Tom Desmet, Pascal Hols, Patrice Soumillion

**Affiliations:** 1 Louvain Institute of Biomolecular Science and Technology, UCLouvain, 1348 Louvain-La-Neuve, Belgium; 2 Department of Biotechnology, Ghent University, 9000 Ghent, Belgium; 3 Biochemistry, University of Otago, PO Box 56, Dunedin, Otago 9054 New Zealand; 4 Department of Biochemistry and Molecular Biology, Michigan State University, East Lansing, MI 48824 USA; 5 Department of Chemistry, Michigan State University, East Lansing, MI 48824 USA; 6 Department of Microbiology and Molecular Genetics, Michigan State University, East Lansing, MI 48824 USA

**Keywords:** Microbiology, Molecular biology, Enzyme mechanisms, Enzymes, Metals

## Abstract

Isomerization reactions are fundamental in biology. Lactate racemase, which isomerizes L- and D-lactate, is composed of the LarA protein and a nickel-containing cofactor, the nickel-pincer nucleotide (NPN). In this study, we show that LarA is part of a superfamily containing many different enzymes. We overexpressed and purified 13 lactate racemase homologs, incorporated the NPN cofactor, and assayed the isomerization of different substrates guided by gene context analysis. We discovered two malate racemases, one phenyllactate racemase, one α-hydroxyglutarate racemase, two D-gluconate 2-epimerases, and one short-chain aliphatic α-hydroxyacid racemase among the tested enzymes. We solved the structure of a malate racemase apoprotein and used it, along with the previously described structures of lactate racemase holoprotein and D-gluconate epimerase apoprotein, to identify key residues involved in substrate binding. This study demonstrates that the NPN cofactor is used by a diverse superfamily of α-hydroxyacid racemases and epimerases, widely expanding the scope of NPN-dependent enzymes.

## Introduction

Chemical isomers exhibit subtle changes in their structures, yet can show dramatic differences in biological properties^1^. Interconversions of isomers by isomerases are important in the metabolism of living organisms and have many applications in biocatalysis, biotechnology, and drug discovery^2^. These enzymes are divided into 6 classes, depending on the type of reaction catalyzed: racemases and epimerases, cis–trans isomerases, intramolecular oxidoreductases, intramolecular transferases, intramolecular lyases, and other isomerases^3^. Only the first category is of interest here.

Racemases and epimerases catalyze the inversion of a stereocenter in molecules containing one (racemases) or several (epimerases) stereocenters. This class is further subdivided into 4 subclasses depending on the substrate used: amino acids and derivatives, α-hydroxyacids and derivatives, carbohydrates and derivatives, and other substrates. This subdivision does not take into account the various chemical strategies employed during catalysis by racemases and epimerases. Indeed, to catalyze inversion of a stereocenter, an epimerase or racemase must break and reform a bond, usually a C-H bond, in a non-stereospecific manner. If the carbon-bound hydrogen atom is sufficiently activated (p*K*_a_ < 30), as for amino acids and mandelic acid, it can be abstracted as a proton, generating an anionic intermediate. The anion derived from amino acids is often stabilized by a pyridoxal phosphate (PLP) cofactor in PLP-dependent amino acid racemases, by a magnesium cation in the case of mandelate racemase, or by the protein in PLP-independent amino acid racemases^4,5^. When the carbon-bound hydrogen atom is not sufficiently activated (p*K*_a_ > 30), as in the case of sugar epimerases, different mechanisms must be used. Most of these enzymes use an NAD cofactor in a transient oxidation, either of a hydroxyl group adjacent to the stereogenic carbon in order to enhance the acidity of the proton, or of the stereogenic carbon itself. Much rarer are examples of cofactor independent epimerases that operate at unactivated stereocenters. UDP-N-acetylglucosamine 2-epimerase and L-ribulose 5-phosphate 4-epimerase are two examples of such epimerases^4^.

**Figure 1:**
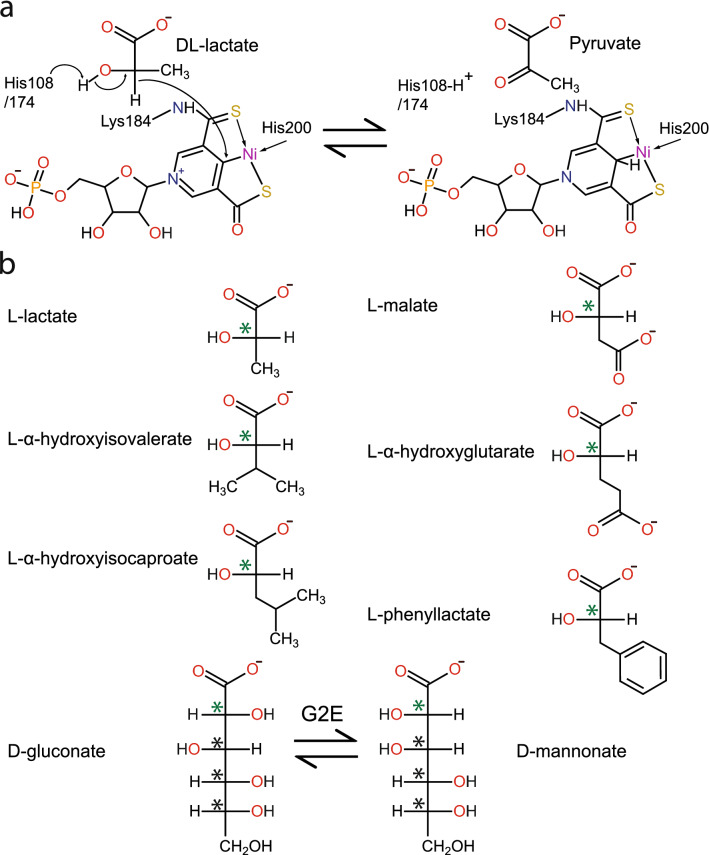
(**a**) NPN cofactor and catalytic mechanism of LarA. (**b**) Substrates identified in the LarA superfamily in Fisher projection. Only one substrate is shown for each pair of substrates, except for G2E. The green asterisks show the sites of stereoinversion, the black asterisks indicate other stereocenters in the molecule.

For lactate racemase (LAR, associated with the *larA* gene product), the stereogenic carbon is transiently oxidized, in this case generating a pyruvate intermediate, similar to the chemistry of UDP-hexose 4-epimerases^6^. Both enzymes catalyze hydride transfer reactions; however, while the hydride is transferred to NAD in UDP-hexose 4-epimerases, it is transferred to a nickel-pincer nucleotide (NPN) cofactor in LAR (Fig. 1a)^7^. The NPN cofactor, discovered in *Lactobacillus plantarum* LarA (hereafter referred to as just LarA)^8^, is synthesized from nicotinic acid adenine dinucleotide using the biosynthetic enzymes LarB, LarE, and LarC which catalyze carboxylation/hydrolysis, sulfur insertion, and nickel insertion reactions, respectively^9–11^. NPN transiently accepts the substrate-derived hydride during the LAR mechanism (Fig. 1a), but it remains unclear whether the hydride is bound only at the C4 position of its pyridinium ring, similar to the case for NAD, or if it may also accept the hydride on its nickel ion; transfer of the hydride between the two sites on the cofactor may facilitate reduction from both sides of the pyruvate intermediate^12^. This particularity of the NPN cofactor may explain why this cofactor is used instead of NAD. Whereas the sugar moiety in UDP-hexose 4-epimerases rotates during its epimerization^6^, rotation of the pyruvate intermediate is unlikely in lactate racemase which thus requires a cofactor able to transfer the hydride from dual positions during racemization.

In this study, we investigated the substrate scope of several homologs of lactate racemase and discovered four new α-hydroxyacid racemases and one new α-hydroxyacid epimerase, thus uncovering a superfamily of NPN-dependent enzymes with various specificities and greatly expanding the number of known nickel enzymes, raising the number from 9^13^ to 14. These enzymes could play important metabolic functions in many microorganisms (no Lar homolog was identified in any multicellular organism) and could also be related to human health and disease by impacting the metabolism of α-hydroxyacids in the gut microbiota.

## Results

### Shedding light on a superfamily of NPN-dependent enzymes

Bacterial and archaeal genes are often clustered into functional units as part of operons^14^ or transcription units^15^, allowing one to infer the purpose of an unknown gene within the cluster^16^. For example, the function of a LarA homolog (LarAH) of *Thermotoga maritima* was postulated to be a D-gluconate 2-epimerase (termed GntE) based on its genomic context^12^. In order to identify the potential functions of other LarAHs, we performed a genomic context analysis with the tool GeConT 2^17^ using the query COG3875. We retrieved sequences encoding the 354 LarAHs from the GeConT 2 database^17^ along with their genomic contexts (3 genes before and 3 genes after the *larAH* gene) and clustered them into 25 phylogenetic groups based on sequence similarities (Fig. S1).

We analyzed the genetic context and listed the most frequently associated COGs (Clusters of Orthologous Groups of proteins) for each LarAH group (Table S1). In 20 out of 25 LarAH groups, we found at least one COG, different from LarB, LarC, or LarE, which showed more than 30% association with LarAH (Table S1), suggesting that these COGs are functionally related with the LarAH group. Some COGs were found in several genomic contexts, e.g. COG0247 or COG 0277, which are groups of oxidoreductases and dehydrogenases, respectively, found in the genomic context of 4 and 5 groups, respectively. Nevertheless, most LarAH groups show a unique genomic context (Table S1), indicating that each LarAH group probably catalyzes a different reaction. From the many different genomic contexts, we identified several possible substrate/LarAH connections; e.g., a nearby gene encoding L-lactate permease suggests a *larA*-like gene encoding another lactate racemase, a malic enzyme could indicate a malate racemase, a sugar kinase or hydratase could indicate a sugar epimerase. In other groups, the context was much less clear and it was not possible to identify a potential substrate (Table S1).

In order to test the reactivity of selected LarAHs with a set of potential substrates, we selected 13 different genes from cultivable bacterial and archaeal species (Fig. S1 and Dataset S1) and constructed vectors to produce the corresponding C-terminal StrepTag fusion proteins for purification from *Escherichia coli* or *Lactococcus lactis*. Among these 13 proteins, 9 were soluble and readily purified (Fig. S2a, b, and c), whereas 4 were insoluble and purified using denaturing conditions (6 M urea) then renatured in buffer without urea (Fig. S2d). Two LarAHs (LarAH3 and LarAH23) showed substantial amounts of contaminating proteins (Fig. S2). Among the 9 soluble LarAHs, three (LarAH6, LarAH7, and LarAH20) were purified from *L. lactis* that coexpressed the NPN-biosynthetic enzymes. Among these, only LarAH7 was significantly loaded with the NPN cofactor after purification, as indicated by the nickel content (~ 10% of the protein; Fig. S3); this value is close to the nickel content of LarA when purified using similar conditions^18^. All remaining LarAHs were supplemented with in vitro synthesized NPN cofactor^10^.

We hypothesized the LarAHs were most likely to catalyze racemization or epimerization of α-hydroxyacids^12^. Based on this hypothesis, on the different genomic contexts (Table S1), and on the availability of assays for the measurement of racemization activities, we tested these purified LarAHs for LAR and several new activities: α-hydroxyisovalerate racemization (HIVR), α-hydroxyisocaproate racemization (HICR), malate racemization (MAR), α-hydroxyglutarate racemization (HGR), D-gluconate 2-epimerization (G2E), and phenyllactate racemization (PLR). A difficulty in studying this family of enzymes is that isomerization reactions generate products very similar to their substrates. This similarity requires that specific enzymatic reactions and/or specific separation techniques be used to detect these conversions. Among the 13 tested LarAHs, we detected one with LAR, HICR, and HIVR activities (termed SAR for short-chain aliphatic α-hydroxyacid racemase activity, hence the enzyme was termed Sar), two with MAR activity (termed Mar), one with HGR activity (termed Hgr), one with PLR activity (termed Plr), and two with G2E activity (termed GntE) (Table 1).

**Table 1:** Activities of LarAHs.

LarA homolog^a^	Species	LAR	HIVR	HICR	MAR	HGR	PLR	G2E
LarA	*Lactobacillus plantarum*	**+**	**−**	**−**	**−**	**−**	**−**	**−**
LarAH2/Sar	*Isosphaera pallida*	**+**	**+**	**+**	**−**	**−**	**−**	**−**
LarAH3^b^	*Streptococcus pneumoniae*	**−**	**−**	**−**	**−**	**−**	**−**	**−**
LarAH4^b^	*Lactobacillus zymae*	**−**	**−**	**−**	**−**	**−**	**−**	**−**
LarAH5/Mar1	*Desulfitobacterium hafniense*	**−**	**−**	**−**	**+**	**−**	**−**	**−**
LarAH6/Mar2	*Thermoanaerobacterium thermosaccharolyticum*	**−**	**−**	**−**	**+**	**−**	**−**	**−**
LarAH7/Hgr	*Deferribacter desulfuricans*	**−**	**−**	**−**	**−**	**+**	**−**	**−**
LarAH8^b^	*Clostridium botulinum*	**−**	**−**	**−**	**−**	**−**	**−**	**−**
LarAH10/Plr	*Megasphaera elsdenii*	**−**	**−**	**−**	**−**	**−**	**+**	**−**
LarAH12^b^	*Candidatus* Nitrososphaera gargensis	**−**	**−**	**−**	**−**	**−**	**−**	**−**
LarAH14	*Frankia* sp.	**−**	**−**	**−**	**−**	**−**	**−**	**−**
LarAH19/GntE1	*Corynebacterium glutamicum*	**−**	**−**	**−**	**−**	**−**	**−**	**+**
LarAH20/GntE2	*Thermotoga maritima*	**−**	**−**	**−**	**−**	**−**	**−**	**+**
LarAH23	*Isosphaera pallida*	**−**	**−**	**−**	**−**	**−**	**−**	**−**

^a^The LarAHX numbers correspond to the numbers of the LarAH groups from Fig. S1.

^b^These enzymes were denatured/renatured.

“+”: activity detected, “−”: activity not detected.

All of these reactions require the NPN cofactor, either added to the assay mix or previously bound to the protein, and lead to the inversion of a stereocenter at the C2 position of an α-hydroxyacid (Fig. 1b), suggesting the enzymes catalyze proton-coupled hydride transfer mechanisms as demonstrated for lactate racemization^7^. The reactions for Mar2 and GntE2 were consistent with their genomic context (Table S1), but such linkage was not apparent for other cases, indicating that the genomic context is not always helpful to determine the activity of an unknown enzyme.

### Biochemical properties of novel isomerases

We characterized the new NPN-dependent activities and compared the properties of these enzymes to those of LarA. First, the optimal pH and apparent optimal temperatures were established (Table 2).

**Table 2:** Kinetic parameters of LarA and 7 LarAHs.

	Substrate	pH opt^a^	Temp opt^a^ (°C)	*K*_M_ (mM)^b^	*k*_cat_ (s^−1^)^b^	*k*_cat_/*K*_M_ (s^−1^ mM^−1^)
LarA^17^	L-lactate	6	35–40	46 ± 20	4,750 ± 500	103 ± 50
	D-lactate			11 ± 4	1,300 ± 150	121 ± 56
LarAH2/Sar	L-lactate	7.5	45–50	0.15 ± 0.04	4.7 ± 0.3	31 ± 10
	D-lactate			0.56 + 0.10	8.1 ± 0.5	14 ± 4
LarAH5/Mar1	L-malate	9	45–50	55 ± 6	140 ± 9	0.39 ± 0.07
LarAH6/Mar2	L-malate	6	35–45	0.38 ± 0.04	66 ± 10	185 ± 49
LarAH7/Hgr	L-hydroxy-glutarate	7–8	NI	0.32 ± 0.06	> 1.5	> 4.7 ± 1
LarAH10/Plr	L-phenyl-lactic acid	NI	NI	0.4 ± 0.3	0.16 ± 0.03	0.28 ± 0.21
LarAH19/GntE1	D-mannonate	5.5–8	30–55	120 ± 23	59 ± 12	0.48 ± 0.15
LarAH20/GntE2	D-mannonate	6	80–90	17 ± 6	197 ± 20	11 ± 5

^a^opt: optimal pH range or temperature (temp), where activity was > 90% of the maximum activity.

^b^±: 95% confidence interval based on non-linear regression using the Michaelis–Menten equation.

NI: not investigated.

Mar1 activity exhibited the highest optimal pH (pH 9), but the significance of this finding is unclear. GntE2 activity from *T. maritima* showed the highest apparent optimal temperature (80–90 °C), consistent with the source being a hyperthermophilic organism.

We characterized the kinetic parameters of the cofactor-supplemented enzymes (Fig. S4 and Table 2) by assuming 100% loading, given that an excess of NPN was added to the apoprotein; however, the extent of NPN loading could not be established. As a result, the values of *k*_cat_ are probably underestimated. Kinetic analysis of Mar2 revealed that it possesses the greatest catalytic efficiency (*k*_cat_/*K*_M_) of 185 ± 49 s^−1^ mM^−1^, whereas GntE2 exhibits the largest *k*_cat_ of 197 ± 20 s^−1^ (Table 2). Hgr, purified after coexpression with the NPN-biosynthetic enzymes and unable to be further activated by added NPN cofactor synthesized in vitro, was very unstable so its reaction rate could not be reliably determined (Table 2). The calculated *k*_cat_ of Plr is very low, potentially suggesting the enzyme was only partially loaded with NPN or very unstable, but it could not be investigated further because of technical reasons (degradation of the capillary coating). Sar (assayed with lactate), Mar2, Hgr, and Plr possess sub-millimolar *K*_M_ values consistent with their use of these substrates in vivo. In contrast, GntE1 and Mar1 possess very high *K*_M_ values (over 50 mM and over 100 mM, respectively), suggesting that the identified substrate may not be the native substrate of these enzymes.

The kinetic parameters of Sar were of interest for substrates other than L- and D-lactate, for which enzymatic assays exist (Table 2). In particular, because we showed this enzyme isomerizes α-hydroxyisovalerate (α-HIV) and α-hydroxyisocaproate (α-HIC) (Table 1) we were interested in the interaction between Sar and D- and L-α-HIV, DL- and L-α-HIC, or related compounds such as L-α-hydroxybutyrate. Therefore, the *K*_i_ of these compounds as competitive inhibitors with L-lactic acid was investigated (Table 3, Fig. S5).

**Table 3:** Sar competitive inhibition constants *K*_i_ for several short-chain aliphatic α-hydroxyacids.

	L-α-hydroxy-butyrate	D-α-hydroxy-isovalerate	L-α-hydroxy-isovalerate	DL-α-hydroxy-isocaproate	L-α-hydroxy-isocaproate
*K*_i_ (mM)	0.31 ± 0.06	0.15 ± 0.02	1.2 ± 0.2	2.1 ± 0.3	14 ± 4

For α-HIV and possibly α-HIC, Sar appears to exhibit greater affinity for the D-isomers (Table 3), rather than the preference for the L-isomer of lactate (Table 2). Moreover, the α-hydroxyacids with the smaller side chains provided *K*_i_ values in the same range as the *K*_M_ of lactate (Table 2 and 3), suggesting a similar affinity. It is likely that α-hydroxybutyrate is also a substrate, consistent with a broad substrate specificity for Sar.

In summary, we characterized seven new NPN-dependent enzymes catalyzing 5 novel α-hydroxyacid isomerizations. We show that at least three of them (Sar, Mar2, and GntE2) demonstrate kinetic parameters expected for enzymes of the LarA superfamily, with catalytic efficiencies in the same range as LarA (Table 2).

### Structural comparison between LarA homologs

**Figure 2:**
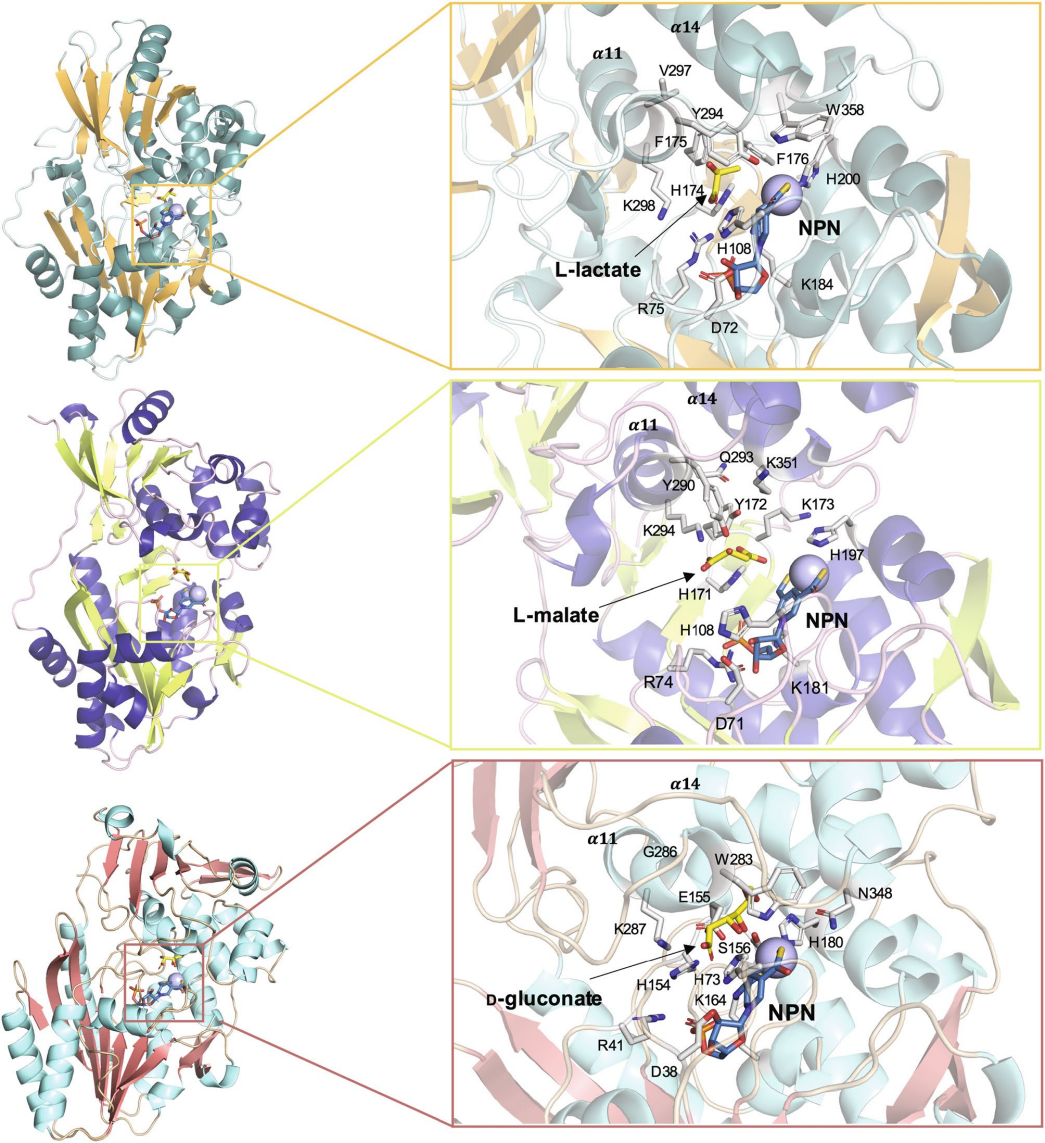
Structural comparison of LarA homologs. Ribbon structures of LarA (top), Mar2 (middle), and GntE2 (bottom) with stick representation used for the residues likely to be involved in catalysis (with C atoms in white, O in red, N in blue, and S in yellow), NPN cofactor (C atoms in light blue), or substrate (C atoms in yellow). The nickel is represented by a light blue ball. Figure drawn with YASARA^20^.

To investigate the basis for substrate specificity of this family of enzymes, we crystallized Mar2 (Table S2) and compared it to the structure of LarA^8^ (Fig. S6). Although Mar2 was produced in *L. lactis* cells coexpressing the NPN-biosynthetic enzymes, the resulting structure lacked the NPN cofactor and substrate. Mar2 possessed the same fold as LarA^8^ that included N-terminal (30–265) and C-terminal (266–415) domains (with the Cα alignments exhibiting an RMSD of 1.05 Å and 1.00 Å, respectively), except for LarA surface loops 94–97 and 241–248, the disordered region ~ 335–358 of Mar2, and the C-terminus of LarA that has the Strep tag modeled; however, unlike the closed and open conformations that differed by a rotational shift of ~ 12° found in LarA^8^, Mar2 possessed a third, more-extended conformation that is rotated by ~ 21° compared to the closed LarA (Fig. S6). A possible interpretation is that Mar2 is in an accessible state that is ready to receive the NPN cofactor. To better compare the Mar2 active site, which is poorly defined in the structure, we used I-Tasser^19^ to model Mar2 using the structure of LarA. We also compared these proteins to the structure of GntE2, previously solved as a protein with unknown function by a structural genomics project (Northeast Structural Genomics Consortium; code 4NAR), which shows a closed conformation lacking cofactor and substrate. To model the presence of the NPN cofactor inside the Mar2 and GntE2 structures, we aligned them with the structure of LarA containing the NPN cofactor using YASARA^20^ and docked the different substrates into the resulting structures (Fig. 2).

We observed that the residues interacting with the NPN cofactor in LarA (R75, K184 and H200) have corresponding residues in Mar2 (R74, K181, and H197) and GntE2 (R41, K164, H180), suggesting that the binding mode for the cofactor is the same in these structures. Similarly, the catalytic residues (D72, H108, H174, and K298 in LarA) are also conserved in the Mar2 and GntE2 active sites, strongly confirming our hypothesis that these enzymes use the same catalytic mechanism as LarA. In contrast, the residues likely to be involved in substrate recognition vary among the LarAH species, consistent with their variable specificities. We identified three regions of the enzymes that are probably involved in substrate recognition based on comparisons of the three structures (Fig. 2). The first region, hereafter named “loop”, is composed of the two residues just after a catalytic histidine (His174 in LarA); these residues are bulky and hydrophobic in LarA (F175 and F176), bulky and positively charged in Mar2 (Y172 and K173), and small polar and negatively charged for GntE2 (E155 and S156), consistent with the substrates they recognize. The second region is located in α-helix 11 (α11), in particular the residue before the catalytic K298 in LarA; this residue is V297 in LarA, a Q293 in Mar2, and G286 in GntE2 (Fig. 2).

The last region potentially involved in substrate binding occurs in the first loop of α-helix 14 (α14), more specifically W358 in LarA, K351 in Mar2, and N348 in GntE2 that point in the direction of the substrate side chain (Fig. 2). Again, in this last region, we observe that a bulky hydrophobic residue in lactate racemase is replaced by a positively charged residue in Mar2, probably binding the second carboxylic acid of malate, and a small residue in GntE2, which has to accommodate a larger substrate. We also observe that α14 angles away from the catalytic site in GntE2, due to the shorter loop between α13 and α14 (Fig. 2). All of these modifications are consistent with these enzymes binding different substrates.

### LarA superfamily organization based on conserved signature motifs

**Figure 3:**
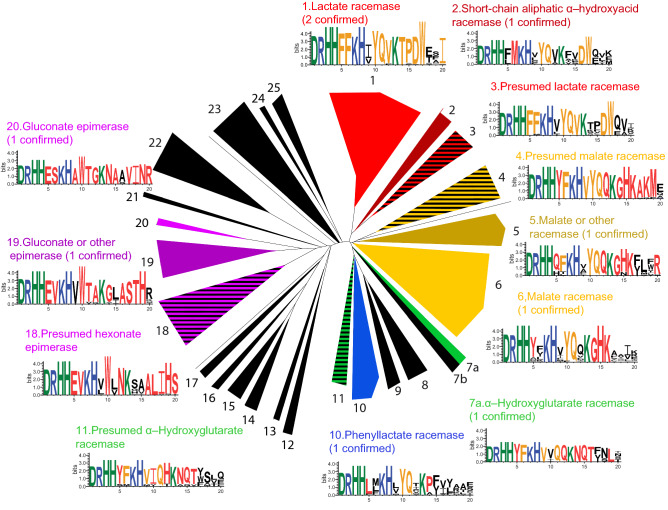
Phylogenetic tree of the LarAH groups. For each group whose activity is suspected the sequence logo is shown, with residues equivalent to D72, R75, H108, H174, F175, F176, K184, H200, I220, Y294, Q295, V297, K298, T353, P355, D356, W358, T359, A360, I362 of LarA (Table S3). Inside each logo, residues involved in catalytic activity are green, those involved in NPN binding are blue, those probably involved in substrate recognition are yellow, conserved residues differing from LarA are red, and those not conserved within the group are black. The logos were generated using Weblogo^21^. The groups for which at least one member have shown enzymatic activity are colored plain, the groups whose activity is suspected are striped black. The groups for which no activity was shown or suspected are colored black.

We created activity signature logos^21^ that incorporate the residues indicated above and other residues which might be involved in substrate selectivity identified from the multiple sequence alignment of the 354 LarAHs (Dataset S2 and Table S3). The LarAH groups, including the newly identified activities, are shown with their corresponding logos in Fig. 3, in order to link signature logos with enzymatic activities. Groups 12, 13, and 16 from the gene context analysis (Table S1) were not included in this analysis because their catalytic and NPN-binding residues were not conserved, suggesting that these enzymes might not be homologs of LarA at all and probably do not use the NPN-cofactor to catalyze their reaction.

Groups 1 and 2, corresponding to LAR and SAR activities, respectively, include several conserved bulky hydrophobic residues associated with the likely substrate-binding region (Fig. 3). Nevertheless, one important difference is the two residues in the loop region, which are both Phe in the LAR group, whereas Leu and Met or Phe and Met are found in the SAR group (Fig. 3). The smaller size of Leu and Met may explain the broader substrate specificity of Sar, compared to LarA. Group 3 shows a signature logo very similar to that of group 1, suggesting this group also contains lactate racemases (Fig. 3).

Groups 5 and 6, comprising Mar1 and Mar2 respectively, show only small differences, as expected. The Phe residue in group 6 is replaced by Gln or His in group 5 (Fig. 3) which indicates a slightly different substrate specificity, consistent with the highly different *K*_M_ values between Mar1 and Mar2 (Table 2). Group 4 shows a signature logo similar to groups 5 and 6 (a GHK motif), suggesting that this group also racemizes malate or a closely related substrate (Fig. 3).

Group 7 was separated into two subgroups, 7a and 7b, due to highly divergent signature logos (Dataset S2). Group 7a, which showed HGR activity, has a similar signature logo as group 11 (including motif NQT, Fig. 3), suggesting that group 11 also racemizes α-hydroxyglutarate. Group 10, which showed PLR activity, has a conserved Leu in the loop region, which may be a signature for PLR activity (Fig. 3). Nevertheless, the rest of the logo is less conserved in this group, which might indicate a larger diversity of substrates. The three groups 18, 19, and 20 show specific features in the loop (EV/S), α11 (W), and α14 (A/V) regions, which suggest similar substrates. Indeed, both groups 19 and 20 were shown to catalyze epimerization of D-mannonate into D-gluconate (Table 1), even if the native substrate for group 19 might be a slightly different molecule, presumably an isomer of D-mannonate, given the high *K*_M_ value of GntE1 for D-mannonate (Table 2) and some differences in the signature logos (Fig. 3). We hypothesize that group 18 also catalyzes the epimerization of a hexonate, similar to groups 19 and 20.

The NPN binding residues and catalytic residues are present in all of the analyzed groups (Fig. 3), strongly suggesting that these LarAHs all bind the NPN cofactor and use the same proton-coupled hydride transfer mechanism as LarA^7^. Some catalytic residues are missing in other groups with unknown substrate specificities, in particular residues corresponding to H174 and K298 in LarA (Table S3). These LarAHs may not be capable of catalyzing the inversion of a stereogenic carbon, but it is conceivable that they could catalyze another type of hydride-mediated reaction.

## Discussion

We described here seven new NPN-dependent enzymes catalyzing five new racemization or epimerization activities involving α-hydroxycarboxylic acids (Table 1). The analysis of the genomic context of the LarAHs helped for the identification of some LarAHs, even if the associated gene only showed 15% association with LarAH (Table 1 LarAH group 6). An important remaining question is the potential roles of these enzymes in vivo. MAR activity was previously observed in *Rhodobacter capsulatus*, but the enzyme was not characterized^22^. This enzyme would allow for growth on D- and L-malate as carbon source without the need for both D- and L-malate dehydrogenases^23^. *Th. thermosaccharolyticum* was not reported to grow on malate, but such a possibility should now be taken into consideration given the identification of Mar2 in this species. Given the low affinity of Mar1 for L-malate (Table 2), one can alternatively hypothesize that malate is not its native substrate. Other similar substrates, like tartrate or 2-hydroxysuccinamate, could be preferentially racemized by this enzyme.

The role of GntE2 was previously hypothesized to convert D-mannonate to D-gluconate^24^, and we have now demonstrated such G2E activity; this reaction probably allows D-mannonate to enter the pentose phosphate pathway^24^. GntE1 also exhibits G2E activity, however its *K*_M_ for D-mannonate is very high (Table 2) and it is part of a distinct LarAH group with a genomic context linked to uronic acid metabolism (Table S1); thus, its reaction might involve the isomerization of a uronic or aldaric acids, e.g. epimerization of D-glucarate to D-mannarate.

The role of Hgr in *D. desulfuricans* is more intriguing, especially considering that it tends to cluster with the molybdenum cofactor biosynthesis enzyme MoaA (Table S1). α-Hydroxyglutarate (α-HG) is a well-known oncometabolite produced from noncanonical enzyme function, especially at acidic pH^25^. In addition, L-α-HG is an intermediate in the lysine degradation pathway of many bacteria^26^. We hypothesize the dual use of D- and L-α-HG as carbon sources by some organisms, with Hgr allowing for the conversion of one isomer into the other that enters a degradation pathway.

Similarly, Sar in *I. pallida* and Plr of *M. elsdenii* probably enables the bacterium to use any isomer of a range of aliphatic α-hydroxyacids as carbon and/or electron sources. Indeed, short-chain aliphatic α-hydroxyacids are produced by *L. lactis* and probably other lactic acid bacteria in anaerobic conditions^27^. Phenyllactate (PLA) also is produced by many *Lactobacillus* species in fermented food products^28^. Considering that *M. elsdenii* consumes lactate produced in the rumen and expresses a lactate racemase^29^ to consume both isomers of this carbon source, it might also need a Plr to racemize PLA and enable the bacterium to use both isomers of this alternative carbon source. 4-Hydroxyphenyllactate, also produced by *Lactobacillus* species^27^, may be an additional substrate of Plr, but this compound could not be tested because enantiomerically pure 4-hydroxyphenyllactate is not commercially available.

Other investigators have demonstrated that LarAHs are ubiquitously expressed within the context of the healthy human gut microbiota^30^, suggesting an important role in this context. Our hypothesis is that D-α-hydroxyacids produced by lactic acid bacteria may have toxic effects in humans and the activity of LarAHs from the microbiome are required for detoxification of these compounds. Two D-α-hydroxyacids have been shown to have adverse effects: D-lactate^31^ and D-hydroxyglutarate^32^, and others probably also show toxicity, as D-hydroxyacids are not usual metabolites of eukaryotic cells.

Most of the studied enzymes, with the notable exception of Sar, showed a very narrow specificity among the substrates tested, each racemizing the stereocenter of a single substrate (Table 1). This observation suggests that each isomerase has evolved for use of a specific substrate and may also explain why multiple LarAHs, up to eight in some cases^12^, are found in a single organism. The specificity of each enzyme requires that distinct proteins be used for the different isomerization reactions. It is likely the cell tightly controls each isomerization reaction to minimize the occurrence of detrimental reactions.

In this study, we discovered five new isomerization activities in the lactate racemase superfamily, demonstrating that the NPN cofactor is used for racemization and epimerization reactions beyond that for lactate. Most of the substrates identified are α-hydroxy acids corresponding to standard α-amino acids (lactate for Ala, α-ΗΙV for Val, α-HIC for Leu, PLA for Phe, malate for Asp, and α-HG for Glu); hence, LarAHs could be involved in amino acid catabolism and/or anabolism. This specificity may also suggest that other corresponding α-hydroxy acids exist and could be substrates of other LarAHs (glycerate, deoxythreonate, 2-hydroxysuccinamate, etc.). The identification of other substrates of LarAHs will require enantiomerically pure compounds, which are not always commercially available, and the use of a separation technique for isomers and enantiomers. Capillary electrochromatography is a potentially useful technique for chiral separation and will be implemented in further studies of LarAHs.

The question then naturally arises whether all LarAH homologs catalyze racemization or epimerization of α-hydroxy acids. The absence of conservation of some critical catalytic residues among selected LarAH groups (Table S3) suggests that additional reactions might occur. In particular, two histidine residues are proposed to act as bases for racemization of lactate depending on the differing orientations of the hydroxyl group for each enantiomer. A LarAH lacking one histidine may use a single enantiomer and transfer the hydride to an adjacent site on the substrate. We expect there to be many other NPN-catalyzed isomerization reactions with broad significance in bacterial metabolism.

## Methods

### Materials and growth conditions

Bacterial strains and plasmids used in the present study are listed in Table S4. Chemicals were purchased from Sigma-Aldrich. *L. lactis* was grown in M17 broth supplemented with 0.5% glucose at 28 °C with chloramphenicol (10 mg·L^−1^). To induce genes under the control of the *nisA* expression signals, nisin A was added during the early exponential phase (OD_600_ = 0.3–0.4) at a concentration of 1 µg·L^−1^ and the cells were collected after 4 h. *E. coli* DH10B cells were grown with agitation at 37 °C in lysogeny broth with ampicillin (200 mg·L^−1^) or erythromycin (200 mg·L^−1^), when required. When expressing pBADHisA derivatives, induction was initiated by addition of L-arabinose (0.2%) and cells were grown at 25 °C for 4 h with agitation.

### DNA techniques

DNA was introduced into *E. coli* and *L. lactis* cells by electrotransformation^33,34^. PCR amplifications used Phusion high-fidelity DNA polymerase (New England Biolabs). The primers used in this study were purchased from Eurogentec and are listed in Table S4. Synthetic genes for all LarAH sequences except LarAH4 and GntE2 were purchased from Biomatik, digested with NcoI and NheI, and cloned into a similarly digested pGIR076^35^ or pGIR072^18^, or into a PciI- and NheI-digested pGIR210 for coexpression with the NPN-biosynthetic enzymes. LarAH4 was amplified (pGIR312_A and B) from the genomic DNA of *L. zymae* purchased from the BCCM collection of microorganisms, digested (PciI and NheI), and cloned into digested (NcoI and NheI) pGIR076. The gene encoding GntE2 was amplified (pGIR212_A and B) from *T. thermotoga* DNA kindly provided from Patrick Wayne, digested (NcoI and NheI), and cloned into digested (PciI and NheI) pGIR210.

### Protein purification and in vitro NPN biosynthesis

In vitro biosynthesis of NPN was performed with purified LarB, LarE, and LarC as previously described^10^. Strep-tagged proteins were purified following published protocols on a Strep-tactin XT column^10^. For purification of insoluble LarAH species, the pellets from 1 L of cell cultures were dissolved in 5 mL of 50 mM Tris–HCl, 50 mM NaCl, 8 M urea, and 10 mM β-mercaptoethanol for 2.5 h at 4 °C with continuous stirring. The solutions were centrifuged at 30,000 × *g* for 30 min at 4 °C and diluted to achieve a concentration of 6 M urea. These solutions were loaded onto Strep-tactin XT columns from IBA Lifesciences and the columns were washed with buffer supplemented with 6 M urea. The purified proteins were eluted with buffer supplemented with 6 M urea. The eluted proteins were ultracentrifuged using Amicon Ultra—4 mL MWCO 10 kDa filter units and diluted with wash buffer until the urea concentration dropped below 20 mM. The identities of each purified LarAH protein were confirmed by electrospray ionization–time-of-flight mass spectrometry analysis. The protein concentrations were estimated with the Bradford assay. The nickel content was estimated using 4-(2-pyridylazo)-resorcinol, as described previously^10^.

### Enzymatic assays

The general protocol for assaying LarAH was as follows: 5 µL biosynthetic NPN-containing mix (NPN approximative final concentration 1 µM) was added to 95 µL reaction mix containing 40 mM substrate (if not otherwise stated), 0.1 µM apoenzyme (final concentration), and the assay for detection of the product (see below) in 100 mM Tris–HCl buffer pH 8. After 5 min incubation, the absorbance at 340 nm was measured every minute with an Infinite 200 PRO plate reader (Tecan). The linear rate of absorbance increase was used for measuring the enzyme rate. Concentrations of the substrate were varied in order to measure *K*_M_. For pH and temperature dependent studies, the reactions were stopped after 10 min by incubation at 90 °C for 5 min then the product concentration was assayed with the detection assay (see below). As GntE2 was not inactivated at 90 °C, we used instead 1 volume of 0.5 M NaOH to stop the reaction. For pH dependence studies, citric acid buffer (pH 2 to pH 4), acetic acid buffer (pH 4 to pH 6), phosphate buffer (pH 6 to pH 8) and borate buffer (pH 8 to pH 10) were used at room temperature. For temperature studies, the enzymes were incubated at the indicated temperatures at pH 8.

The LAR assay used the D-/L-Lactic Acid Assay Kit from Megazyme as described^18^ with L-lactate as substrate.

For the HIVR and HICR assays with 40 mM L-HIV or L-HIC as substrate, the D-HIV or D-HIC was assayed with D-hydroxyacid dehydrogenase PanE, as described^27^.

For the MAR assay with L-malate as substrate, *E. coli* D-malate dehydrogenase was purified as described^36^ and 5 µM was used along with L-malate in the presence of 1 mM of NAD, 1 M KCl, and 0.1 M Tris–HCl buffer, pH 8.

The HGR assay used L-α-HG as substrate and *Acidaminococcus fermentans* D-hydroxyglutarate dehydrogenase (HGDH)^37^; for the latter, the synthetic gene was purchased from Biomatik, cloned into pBADHisA (NcoI, HindII), and purified as previously described using a Strep-tactin XT column^10^. The D-α-HG assay was performed with 5 µM purified HGDH, 1% V/V glutamate-pyruvate transaminase from the D-/L-Lactic Acid Assay Kit from Megazyme, 1 mM NAD, 0.1 M D-alanine, and 0.1 M Tris–HCl buffer, pH 8.

The G2E assay used D-mannonate as substrate and the D-gluconate Assay Kit from Megazyme.

The PLR assay used L-phenyllactate as substrate with the reaction mixture stopped after 5 min by incubation at 90 °C, ultrafiltered using an Amicon Ultra—0.5 mL MWCO 10 kDa filter unit, loaded onto a PVA-coated capillary of 55 cm length with an internal diameter of 50 μm from Agilent, and run for 50 min on a Capel 105 M from Lumex Instrument at 20 °C using -25 kV. The background electrolyte was 50 mM phosphate buffer, pH 6, with 125 mM β-cyclodextrin as chiral separator. Products were detected at 190 nm.

### Crystallization

The C-terminal Strep-tag II version of Mar2 was purified from *L. lactis*, concentrated by centrifugal filtration, and subjected to size exclusion chromatography (S200) using resin that was equilibrated with 100 mM Tris–HCl, pH 7.5, containing 300 mM NaCl. We observed a single peak consistent with a monomer, shown by comparing to molecular weight standards. 2 μL samples of concentrated Mar2 at ~ 12 mg/ml (estimated by the Bradford assay) were mixed with 1 μL of reservoir solution. The sitting drop reservoir contained 100 μl of 0.2 M (NH_4_)_2_SO_4_, 0.1 M Bis–Tris, pH 5.5, and 25% w/v polyethylene glycol 3,350. Crystals appeared and fully grew as thin needles within two weeks. The crystals were soaked for about two min in 0.15 M (NH_4_)_2_SO_4_, 0.075 M Bis–Tris, pH 5, and 32% w/v polyethylene glycol 3,350 prior to freezing in liquid nitrogen. Data collection and refinement statistics are provided in Table S2. Data were processed with xdsapp^38^, with merging and scaling performed using aimless^39^. Molecular replacement^40^ used two search models of individual domains of LarA (PDB ID 5HUQ). Model building and refinement were conducted in Coot^41^ and Phenix^40^. The structure was deposited under PDB ID 6D6Z. UCSF Chimera^42^ was used to create structure figures.

### In silico* analyzes*

The multiple alignment was performed using ClustalX^43^ and the phylogenetic tree was visualized using Dendroscope 3^44^. The residues selected for analysis (Fig. 3) were selected based on visual inspection of the structures of LarA, Mar2, and Gnte2 using Pymol^45^.

With the exception of NPN, all ligands were retrieved from the PubChem database and imported into YASARA (Yet Another Scientific Artificial Reality Application, www.yasara.org) for file conversion^20^. Crystal structures of the different Lar homologues were prepared by performing optimizations of the hydrogen bonding networks and energy minimizations using the YASARA force field (derived from the AMBER force field). A simulation box for docking was defined at 8 Å around the crucial residues. The binding of the α-hydroxyacid ligands was simulated using the implemented AutoDock VINA module^46^ with default parameters, except for the number of runs which was increased to 100. The most accurate models were selected on the basis of the best binding energies and known interactions.

For the modeling of Mar2, I-tasser^19^ was used with the default parameters and the sequence of Mar2 as entry. The Model1 was chosen as it fitted best with LarA. The crystal structure of LarA (PDB: 5HUQ) bearing the cofactor was used as the template for the docking of NPN into the GntE and Mar2 structures. To do so, a multiple structural alignment using the built-in MUSTANG module^47^ was performed. Next, the enzyme structure used as the template was removed, while allowing the NPN to be coordinated by the residues of the homologues.

Figures were made with PyMOL v1.3^45^.

## Supplementary information

Supplementary file1

Supplementary file2
