# Supplementary material for: Uncovering a superfamily of nickel-dependent hydroxyacid racemases and epimerases
Source: Sci Rep. 2020 Oct 22;10:18123. doi: 10.1038/s41598-020-74802-6 (PMC7583248; doi:10.1038/s41598-020-74802-6)
Supplement: Supplementary file 2 — Supplementary file2 [file 41598_2020_74802_MOESM2_ESM.docx]

**Supplementary Information for**

Uncovering a superfamily of nickel-dependent hydroxyacid racemases and epimerases

**Benoît Desguin*****, Julian Urdiain-Arraiza, Matthieu Da Costa, Matthias Fellner, Jian Hu, Robert P. Hausinger, Tom Desmet, Pascal Hols, and Patrice Soumillion**

*Corresponding author: Benoît Desguin

**Email:**  [benoit.desguin@uclouvain](mailto:benoit.desguin@uclouvain%20)

**Other supplementary materials for this manuscript include the following:**

Datasets S1 to S2


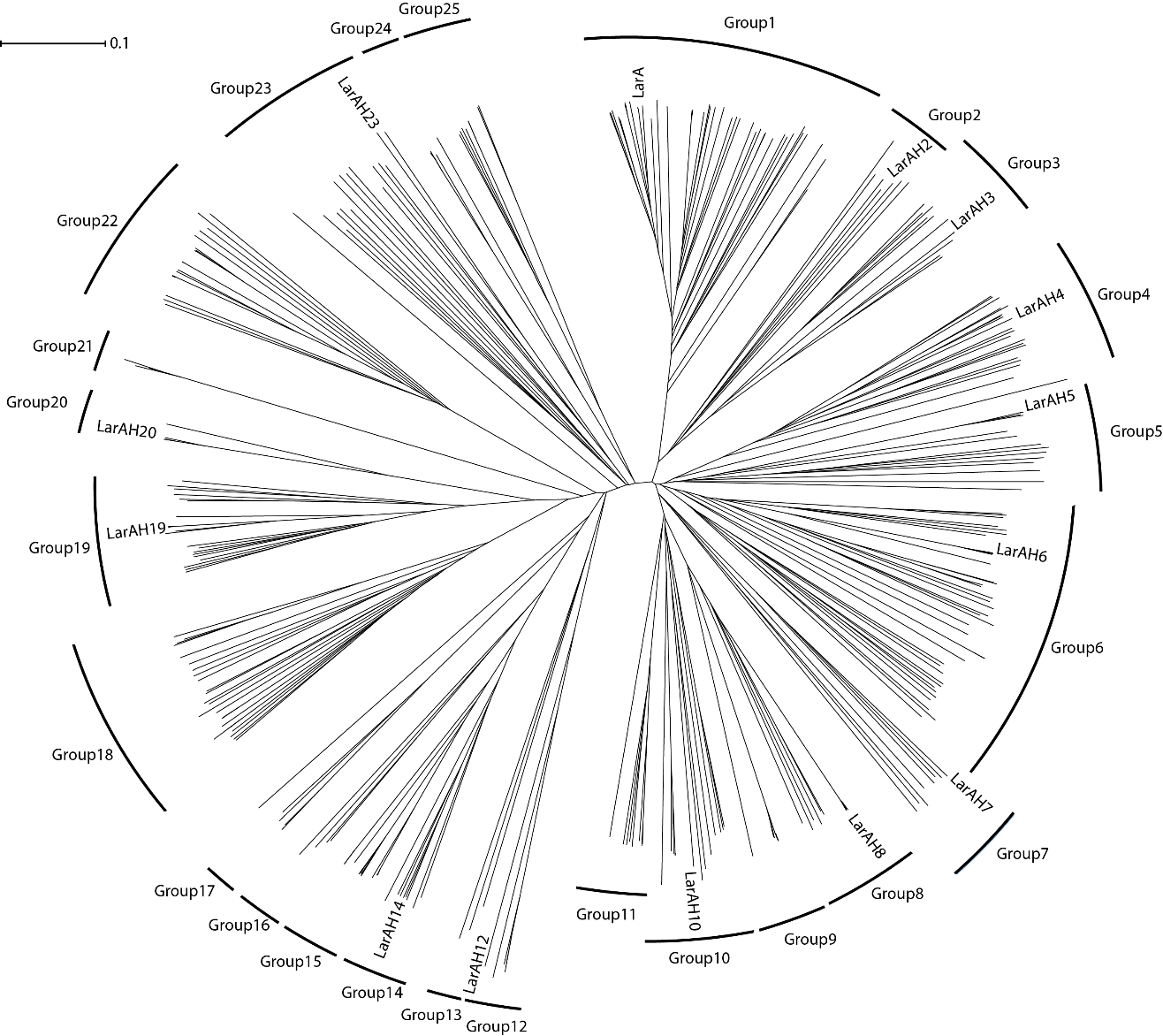


**Figure S1.** Phylogenetic tree of 354 LarAHs and clustering into 25 groups. LarA and 13 LarAH proteins selected for purification are shown. Figure drawn using Dendroscope (1).

**
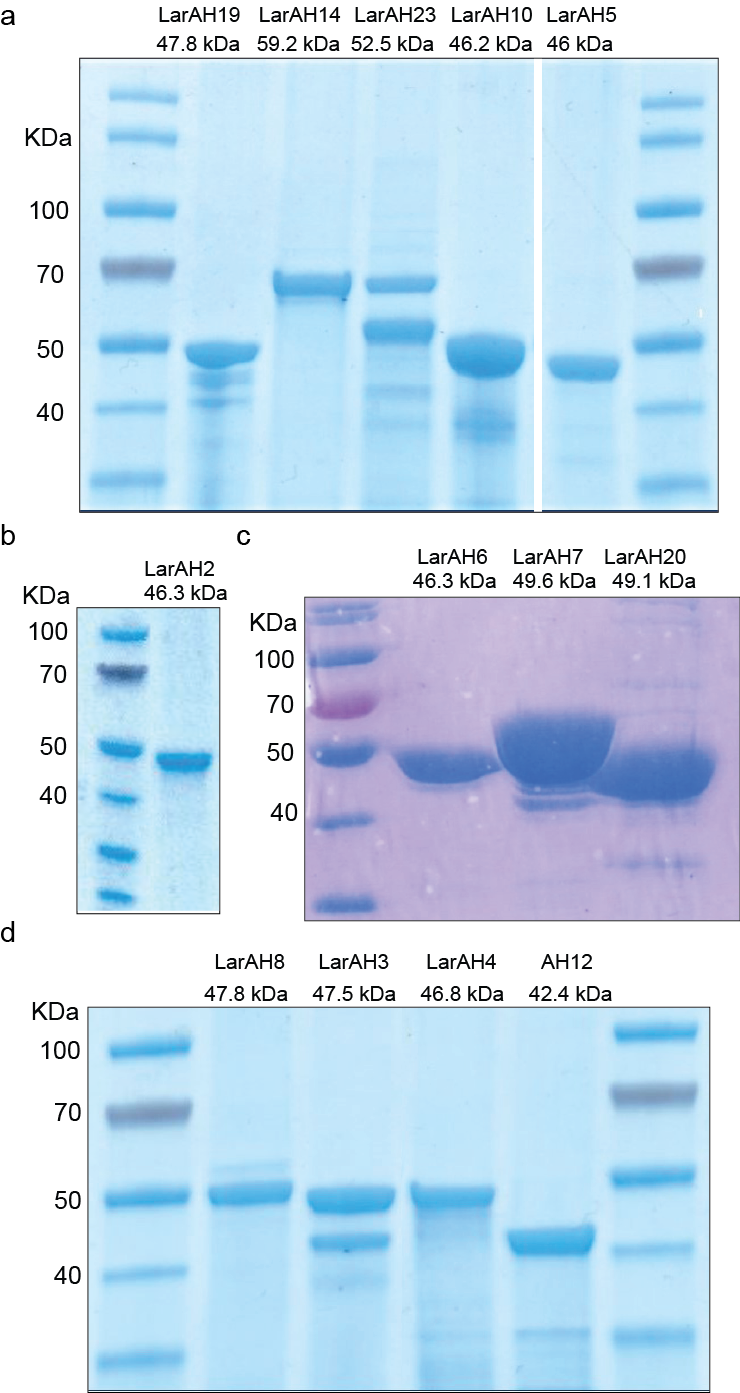
**

**Figure S2.** Analysis of purified LarAHs by SDS-PAGE. **a** and **b**, soluble LarAHs purified from *E. coli*. **c**, soluble LarAHs purified from *Lc. lactis*. **d**, insoluble LarAHs purified from *E. coli* using denaturing conditions. The sequences of these proteins are provided in Dataset S2.

**
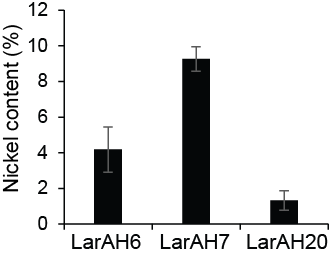
**

**Figure S3.** Nickel content of purified enzymes from *Lc. lactis* cells that also synthesized LarB, LarC, LarD, and LarE. The error bars represent the standard deviation (n=3).


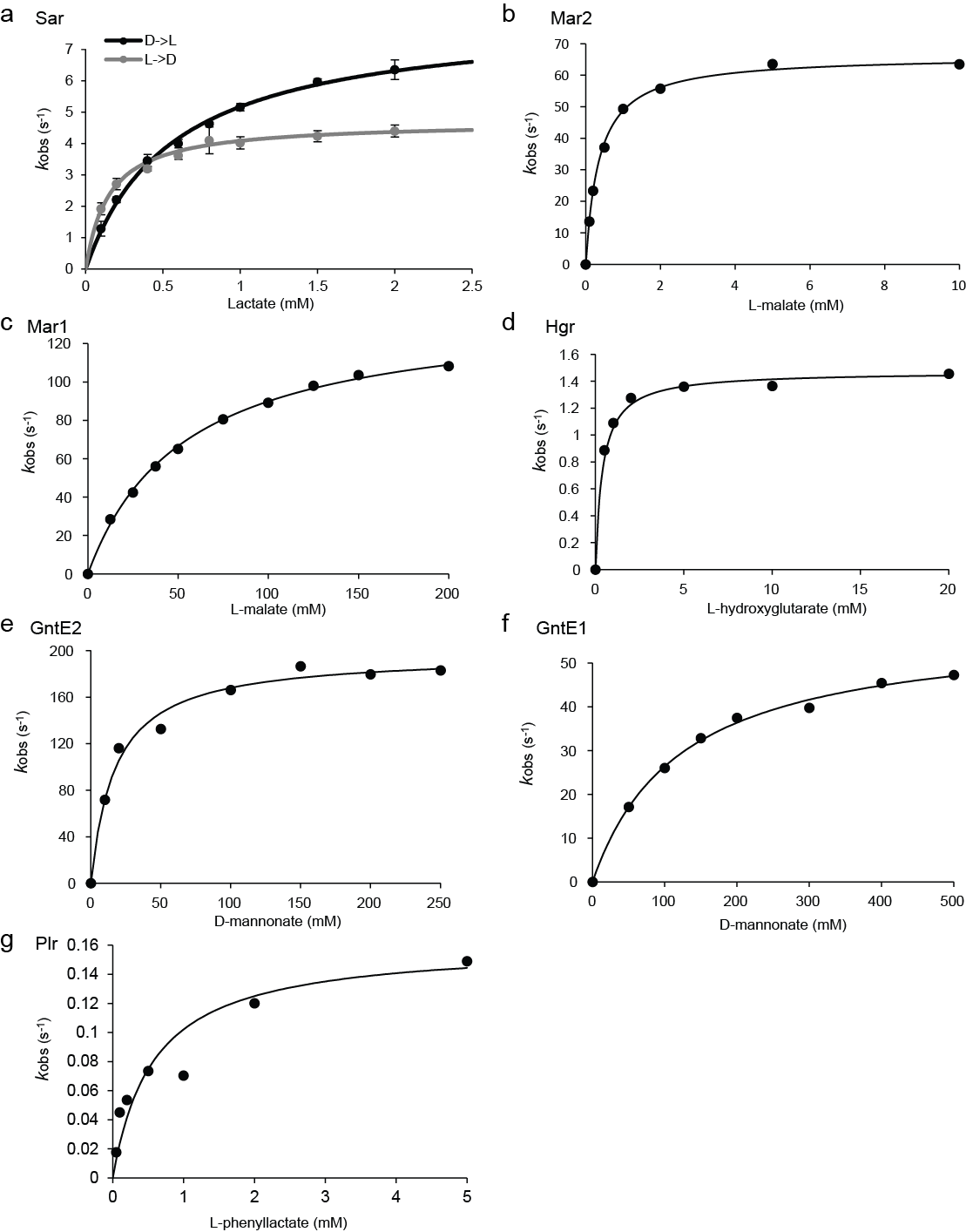


**Figure S4.** Kinetic analyses of NPN-dependent enzymes. **a**, short-chain aliphatic α-hydroxyacid racemase of *Isosphaera pallida* (LarAH2/Sar). **b**, malate racemase (LarAH6/Mar2) from *Thermoanaerobacterium thermosaccharolyticum*. **c**, malate racemase (Mar1 or LarAH5) from *Desulfitobacterium hafniense*. **d**, hydroxyglutarate racemase (LarH7/Hgr) from *Deferribacter desulfuricans*. **e**, gluconate-mannonate epimerase (LarH20/GntE2) from *Thermotoga maritima*. **f**, gluconate-mannonate epimerase (LarAH19/GntE1) from *Corynebacterium glutamicum*. **g**, phenyllactate racemase (LarAH10/Plr) from *Megasphaera elsdenii*. The activities of the enzymes at the indicated substrate concentrations are shown as *k*_obs_ = *v*_0_/(E)_0_. The curves in the kinetic studies are the fitted curves using non-linear regression based on the Michaelis-Menten equation.


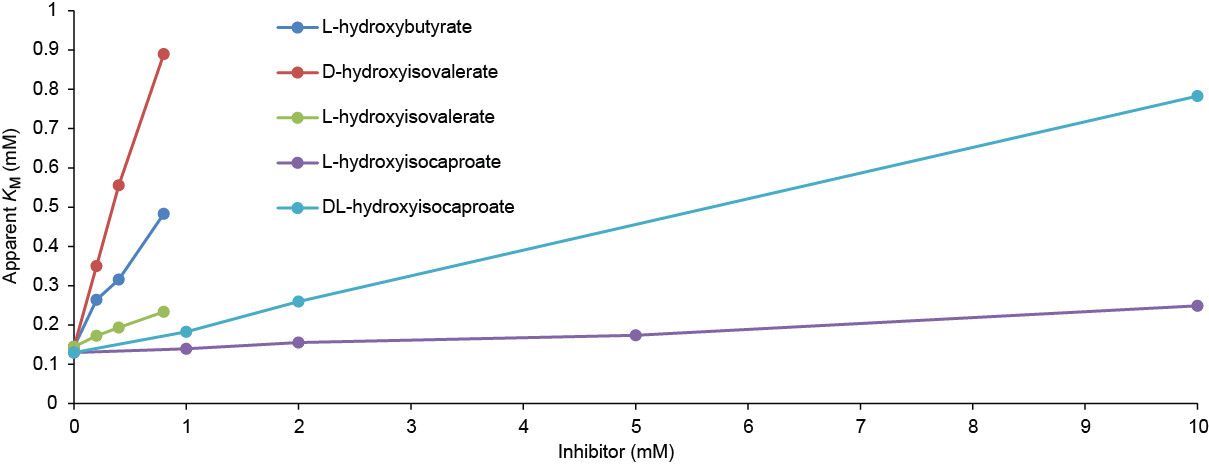


**Figure S5.** Competitive inhibition studies of Sar activity on L-lactate by several short-chain aliphatic α-hydroxyacids.


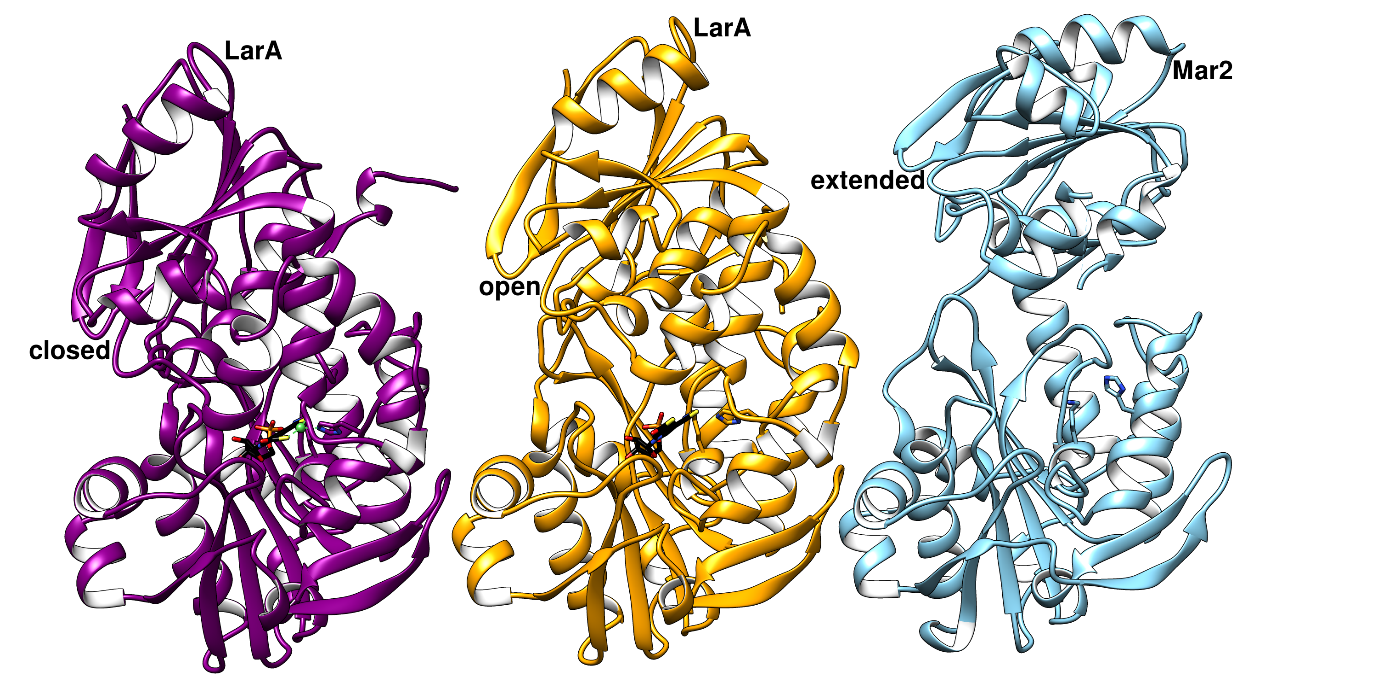


**Figure S6.** Alignment of the N-terminal domain (residues 38-266) of LarA PDB ID 5HUQ in its closed and open conformations with the corresponding domain (residues 37-265) of Mar2 PDB ID 6D6Z), showing movement of the C-terminal domain (LarA 267-424, Mar2 266-417). The figures were drawn using Chimera (2).

**Table S1.** Genomic context of each LarAH homolog group.

| **LarAH**  **homolog**  **group** | **# Genes** | **% as-socia-tion** | **COG** | **Probable function** |
| --- | --- | --- | --- | --- |
| Group1 |  |  |  |  |
|  | 57 | 100 | COG3875 | LarA |
|  | 24 | 42 | COG1641 | LarC |
|  | 20 | 35 | COG0277 | FAD/FMN-containing dehydrogenase |
|  | 19 | 33 | COG1691 | LarB |
|  | 19 | 33 | COG2086 | Electron transfer flavoprotein, alpha and beta subunits |
|  | 18 | 32 | COG2025 | Electron transfer flavoprotein, alpha subunit |
|  | 15 | 26 | COG0664 | cAMP-binding domain of CRP or a regulatory subunit of cAMP-dependent protein kinases |
|  | 14 | 25 | COG1620 | L-lactate permease |
|  | 10 | 18 | COG0310 | ABC-type Co2+ transport system, permease component |
|  | 10 | 18 | COG0580 | LarD |
|  | 10 | 18 | COG0619 | Energy-coupling factor transporter transmembrane protein EcfT |
| Group2 |  |  |  |  |
|  | 7 | 100 | COG3875 | LarAH |
| Group3 |  |  |  |  |
|  | 14 | 100 | COG3875 | LarAH |
|  | 9 | 64 | COG1606 | LarE |
|  | 9 | 64 | COG1691 | LarB |
|  | 5 | 36 | COG0179 | 2-keto-4-pentenoate hydratase/2-oxohepta-3-ene-1,7-dioic acid hydratase (catechol pathway) |
|  | 5 | 36 | COG1641 | LarC |
|  | 5 | 36 | COG3618 | Predicted metal-dependent hydrolase, TIM-barrel fold |
|  | 4 | 29 | COG2378 | Predicted DNA-binding transcriptional regulator YafY, contains an HTH and WYL domains |
|  | 4 | 29 | COG4948 | L-alanine-DL-glutamate epimerase or related enzyme of enolase superfamily |
| group4 |  |  |  |  |
|  | 18 | 100 | COG3875 | LarAH |
|  | 7 | 39 | COG0247 | Fe-S oxidoreductase |
|  | 5 | 28 | COG1606 | LarE |
|  | 5 | 28 | COG1641 | LarC |
|  | 5 | 28 | COG1691 | LarB |
|  | 3 | 17 | COG0126 | 3-phosphoglycerate kinase |
|  | 3 | 17 | COG0277 | FAD/FMN-containing dehydrogenase |
|  | 3 | 17 | COG0294 | Dihydropteroate synthase |
|  | 3 | 17 | COG1377 | Flagellar biosynthesis protein FlhB |
|  | 3 | 17 | COG1908 | Coenzyme F420-reducing hydrogenase, delta subunit |
| group5 |  |  |  |  |
|  | 19 | 100 | COG2721 | Altronate dehydratase |
|  | 15 | 79 | COG3875 | LarAH |
|  | 5 | 26 | COG3181 | Tripartite-type tricarboxylate transporter, receptor component TctC |
|  | 5 | 26 | COG3333 | TctA family transporter |
|  | 3 | 16 | COG3604 | Transcriptional regulator containing GAF, AAA-type ATPase, and DNA-binding Fis domains |
|  | 3 | 16 | COG3679 | Cell fate regulator YlbF, YheA/YmcA/DUF963 family (controls sporulation, competence, biofilm development) |
| group6 |  |  |  |  |
|  | 41 | 100 | COG3875 | LarAH |
|  | 6 | 15 | COG0281 | Malic enzyme |
|  | 5 | 12 | COG0179 | 2-keto-4-pentenoate hydratase |
|  | 5 | 12 | COG0247 | Fe-S oxidoreductase |
|  | 5 | 12 | COG0277 | FAD/FMN-containing dehydrogenase |
|  | 4 | 10 | COG1236 | RNA processing exonuclease, beta-lactamase fold, Cft2 family |
|  | 4 | 10 | COG2876 | 3-deoxy-D-arabino-heptulosonate 7-phosphate (DAHP) synthase |
|  | 3 | 7 | COG0265 | Periplasmic serine protease, S1-C subfamily, contain C-terminal PDZ domain |
|  | 3 | 7 | COG0479 | Succinate dehydrogenase/fumarate reductase, Fe-S protein subunit |
|  | 3 | 7 | COG1151 | Hydroxylamine reductase (hybrid-cluster protein) |
|  | 3 | 7 | COG1309 | DNA-binding transcriptional regulator, AcrR family |
| group7 |  |  |  |  |
|  | 8 | 100 | COG3875 | LarAH |
|  | 4 | 50 | COG0805 | Sec-independent protein secretion pathway component TatC |
|  | 4 | 50 | COG1826 | Sec-independent protein translocase protein TatA |
|  | 4 | 50 | COG2896 | Molybdenum cofactor biosynthesis enzyme MoaA |
| group8 |  |  |  |  |
|  | 15 | 100 | COG3875 | LarAH |
|  | 9 | 60 | COG1187 | 16S rRNA U516 pseudouridylate synthase RsuA and related 23S rRNA U2605, pseudouridylate synthases |
|  | 9 | 60 | COG1215 | Glycosyltransferase, catalytic subunit of cellulose synthase and poly-beta-1,6-N-acetylglucosamine synthase |
|  | 9 | 60 | COG1737 | DNA-binding transcriptional regulator, MurR/RpiR family, contains HTH and SIS domains |
|  | 8 | 53 | COG0334 | Glutamate dehydrogenase/leucine dehydrogenase |
|  | 8 | 53 | COG3681 | L-cysteine desulfidase |
| group9 |  |  |  |  |
|  | 10 | 100 | COG3875 | LarAH |
|  | 9 | 90 | COG0075 | Archaeal aspartate aminotransferase or a related aminotransferase, includes purine catabolism protein PucG |
|  | 9 | 90 | COG4845 | Chloramphenicol O-acetyltransferase |
|  | 8 | 80 | COG0765 | ABC-type amino acid transport system, permease component |
|  | 8 | 80 | COG0834 | ABC-type amino acid transport/signal transduction system, periplasmic component/domain |
|  | 8 | 80 | COG1126 | ABC-type polar amino acid transport system, ATPase component |
|  | 8 | 80 | COG3478 | Predicted nucleic-acid-binding protein, contains Zn-ribbon domain |
| group10 |  |  |  |  |
|  | 13 | 100 | COG3875 | LarAH |
|  | 4 | 31 | COG0277 | FAD/FMN-containing dehydrogenase |
|  | 3 | 23 | COG0119 | Isopropylmalate/homocitrate/citramalate synthases |
|  | 3 | 23 | COG0247 | Fe-S oxidoreductase |
|  | 3 | 23 | COG0538 | Isocitrate dehydrogenase |
|  | 3 | 23 | COG0591 | Na^+^/proline symporter |
|  | 3 | 23 | COG0840 | Methyl-accepting chemotaxis protein |
| group11 |  |  |  |  |
|  | 8 | 100 | COG3875 | LarAH |
|  | 8 | 100 | COG0247 | Fe-S oxidoreductase |
|  | 8 | 100 | COG0277 | FAD/FMN-containing dehydrogenase |
|  | 5 | 63 | COG1396 | Transcriptional regulator, contains XRE-family HTH domain |
|  | 5 | 63 | COG3808 | Na^+^ or H^+^-translocating membrane pyrophosphatase |
|  | 3 | 38 | COG0297 | Glycogen synthase |
|  | 3 | 38 | COG0426 | Flavorubredoxin |
|  | 3 | 38 | COG0501 | Zn-dependent protease with chaperone function |
|  | 3 | 38 | COG1592 | Rubrerythrin |
|  | 3 | 38 | COG1633 | Rubrerythrin |
|  | 3 | 38 | COG4799 | Acetyl-CoA carboxylase, carboxyltransferase component |
| group12 |  |  |  |  |
|  | 5 | 100 | COG3875 | LarAH |
|  | 5 | 100 | COG0456 | Ribosomal protein S18 acetylase RimI and related acetyltransferases |
|  | 5 | 100 | COG0461 | Orotate phosphoribosyltransferase |
|  | 4 | 80 | COG1994 | Zn-dependent protease (includes SpoIVFB) |
|  | 3 | 60 | COG5424 | Pyrroloquinoline quinone (PQQ) biosynthesis protein C |
|  | 3 | 60 | COG5625 | Predicted DNA-binding transcriptional regulator, contains HTH domain |
| group13 |  |  |  |  |
|  | 3 | 100 | COG3875 | LarAH |
| group14 |  |  |  |  |
|  | 8 | 100 | COG3875 | LarAH |
|  | 7 | 88 | COG0204 | 1-acyl-sn-glycerol-3-phosphate acyltransferase |
|  | 7 | 88 | COG0560 | Phosphoserine phosphatase |
|  | 7 | 88 | COG1063 | Phosphoserine phosphatase |
|  | 4 | 50 | COG3320 | Thioester reductase domain of alpha aminoadipate reductase Lys2 and NRPSs |
|  | 3 | 38 | COG0258 | 5'-3' exonuclease |
|  | 3 | 38 | COG0451 | Nucleoside-diphosphate-sugar epimerase |
| group15 |  |  |  |  |
|  | 12 | 100 | COG3875 | LarAH |
|  | 18 | 150 | COG0451 | Nucleoside-diphosphate-sugar epimerase |
|  | 12 | 100 | COG0560 | Phosphoserine phosphatase |
|  | 9 | 75 | COG1022 | Long-chain acyl-CoA synthetase (AMP-forming) |
|  | 4 | 33 | COG5531 | Chromatin remodeling complex protein RSC6, contains SWIB domain |
| group16 |  |  |  |  |
|  | 4 | 100 | COG3875 | LarAH |
|  | 4 | 100 | COG0204 | 1-acyl-sn-glycerol-3-phosphate acyltransferase |
|  | 4 | 100 | COG0560 | Phosphoserine phosphatase |
| group17 |  |  |  |  |
|  | 4 | 100 | COG3875 | LarAH |
|  | 3 | 75 | COG0149 | Triosephosphate isomerase |
|  | 2 | 50 | COG0057 | Glyceraldehyde-3-phosphate dehydrogenase/erythrose-4-phosphate dehydrogenase |
| group18 |  |  |  |  |
|  | 25 | 100 | COG3875 | LarAH |
|  | 9 | 36 | COG1028 | NAD(P)-dependent dehydrogenase, short-chain alcohol dehydrogenase family |
|  | 8 | 32 | COG0362 | 6-phosphogluconate dehydrogenase |
|  | 4 | 16 | COG0524 | Sugar or nucleoside kinase, ribokinase family |
|  | 3 | 12 | COG0477 | MFS family permease |
|  | 3 | 12 | COG1904 | Glucuronate isomerase |
|  | 3 | 12 | COG3717 | 5-keto 4-deoxyuronate isomerase |
| group19 |  |  |  |  |
|  | 21 | 100 | COG3875 | LarAH |
|  | 7 | 33 | COG1606 | LarE |
|  | 7 | 33 | COG1691 | LarB |
|  | 5 | 24 | COG1609 | DNA-binding transcriptional regulator, LacI/PurR family |
|  | 5 | 24 | COG1904 | Glucuronate isomerase |
|  | 4 | 19 | COG0246 | Mannitol-1-phosphate/altronate dehydrogenases |
|  | 4 | 19 | COG1028 | NAD(P)-dependent dehydrogenase, short-chain alcohol dehydrogenase family |
|  | 4 | 19 | COG1641 | LarC |
|  | 3 | 14 | COG1335 | Nicotinamidase-related amidase |
|  | 3 | 14 | COG2141 | Flavin-dependent oxidoreductase, luciferase family (includes alkanesulfonate monooxygenase SsuD and methylene tetrahydromethanopterin reductase) |
|  | 3 | 14 | COG3250 | Beta-galactosidase/beta-glucuronidase |
| group20 |  |  |  |  |
|  | 7 | 100 | COG3875 | LarAH |
|  | 7 | 100 | COG1028 | NAD(P)-dependent dehydrogenase, short-chain alcohol dehydrogenase family |
|  | 6 | 86 | COG1027 | Aspartate ammonia-lyase |
|  | 6 | 86 | COG1070 | Sugar (pentulose or hexulose) kinase |
|  | 6 | 86 | COG1160 | Predicted GTPases |
|  | 6 | 86 | COG1802 | DNA-binding transcriptional regulator, GntR family |
| group21 |  |  |  |  |
|  | 6 | 100 | COG3875 | LarAH |
|  | 6 | 100 | COG0679 | Predicted permease |
|  | 6 | 100 | COG1366 | Anti-anti-sigma regulatory factor (antagonist of anti-sigma factor) |
|  | 6 | 100 | COG2172 | Anti-sigma regulatory factor (Ser/Thr protein kinase) |
|  | 6 | 100 | COG2208 | Serine phosphatase RsbU, regulator of sigma subunit |
|  | 6 | 100 | COG2770 | HAMP domai |
|  | 5 | 83 | COG1653 | ABC-type glycerol-3-phosphate transport system, periplasmic component |
| group22 |  |  |  |  |
|  | 20 | 100 | COG3875 | LarAH |
|  | 9 | 45 | COG1028 | NAD(P)-dependent dehydrogenase, short-chain alcohol dehydrogenase family |
|  | 6 | 30 | COG0129 | Dihydroxyacid dehydratase/phosphogluconate dehydratase |
|  | 6 | 30 | COG1606 | LarE |
|  | 6 | 30 | COG1638 | TRAP-type C4-dicarboxylate transport system, periplasmic component |
|  | 6 | 30 | COG3090 | TRAP-type C4-dicarboxylate transport system, small permease component |
|  | 5 | 25 | COG1070 | Sugar (pentulose or hexulose) kinase |
|  | 5 | 25 | COG1414 | DNA-binding transcriptional regulator, IclR family |
|  | 5 | 25 | COG1593 | TRAP-type C4-dicarboxylate transport system, large permease component |
|  | 4 | 20 | COG1904 | Glucuronate isomerase |
|  | 4 | 20 | COG1940 | Sugar kinase of the NBD/HSP70 family, may contain an N-terminal HTH domain |
| group23 |  |  |  |  |
|  | 15 | 100 | COG3875 | LarAH |
| group24 |  |  |  |  |
|  | 5 | 100 | COG3875 | LarAH |
|  | 3 | 60 | COG0375 | Zn finger protein HypA/HybF (possibly regulating hydrogenase expression) |
|  | 3 | 60 | COG0378 | Ni^2+^-binding GTPase involved in regulation of expression and maturation of urease and hydrogenase |
|  | 3 | 60 | COG0436 | Aspartate/methionine/tyrosine aminotransferase |
|  | 3 | 60 | COG0474 | Magnesium-transporting ATPase (P-type) |
|  | 3 | 60 | COG1595 | DNA-directed RNA polymerase specialized sigma subunit, sigma24 family |
|  | 3 | 60 | COG3794 | Plastocyanin |
| group25 |  |  |  |  |
|  | 8 | 100 | COG3875 | LarAH |
|  | 4 | 50 | COG1112 | Superfamily I DNA and/or RNA helicase |

**Table S2.** Crystal statistics for the Mar2 structure.

| **Data collection** | **Mar2** |
| --- | --- |
| Beamline | LS-CAT 21-ID-D |
| Wavelength (Å) | 1.078 |
| Space group | I 2 2 2 |
| Unit cell a, b, c (Å); α, β, γ (°) | 73 103 134; 90, 90, 90 |
| ^a^Resolution (Å) | 44.41 – 2.38 (2.46 – 2.38) |
| Unique reflections | 20,224 (1,910) |
| ^a^Redundancy | 9.9 (9.4) |
| ^a^Completeness (%) | 98.3 (89.7) |
| ^a^*I/σI* | 12.3 (2.3) |
| ^a,b^*R_merge_* | 0.119 (0.947) |
| ^a,c^*R_pim_* | 0.058 (0.465) |
| ^d^CC_1/2_ | 0.998 (0.844) |
| **Data refinement** |  |
| Protein atoms | 2,975 |
| H_2_O molecules | 93 |
| ^e^*R_work_/R_free_* | 0.183/0.239 |
| *B*-factors (Å^2^) | 50.1 |
| Protein atoms | 50.2 |
| H_2_O molecules | 44.7 |
| R.m.s. deviation in bond lengths (Å) | 0.008 |
| R.m.s. deviation in bond angles (°) | 0.887 |
| Ramachandran plot (%) favored | 96.4 |
| Ramachandran plot (%) outliers | 0 |
| Rotamer outliers | 0 |
| PDB ID | 6D6Z |

^a^Highest resolution shell is shown in parentheses.

^b^*R_merge_* = ∑*_hkl_* ∑*_j_* |*I_j_*(*hkl*)-<I(*hkl*)>| / ∑_hkl_ ∑*_j_* *I_j_*(*hkl*), where *I* is the intensity of reflection.

^c^*R_pim_*=∑*_hkl_* [1/(N-1)]^1/2^∑*_j_* |*I_j_*(*hkl*)-<I(*hkl*)>| / ∑*_hkl_* ∑*_j_* *I_j_*(*hkl*), where N is the redundancy of the dataset.

^d^CC_1/2_ is the correlation coefficient of the half datasets.

^e^*R_work_* = ∑*_hkl_* | |*F_obs_*| – |*F_calc_*| | / ∑*_hkl_* |*F_obs_*|, where *F_obs_* and *F_calc_* is the observed and the calculated structure factor, respectively. R_free_ is the cross-validation R factor for the test set of reflections (10% of the total) omitted in model refinement.

**Table S3.** For all LarAH groups, the logo is shown for the residues equivalent to D72, R75, H108, H174, F175, F176, K184, H200, I220, Y294, Q295, V297, K298, T353, P355, D356, W358, T359, A360, I362 of LarA.

| **LarAH group** | **Logo of selected residues (Fig. 3)**^a^  **Loop α11 α14** | **#^b^** | **Function** |
| --- | --- | --- | --- |
| Group 1 | 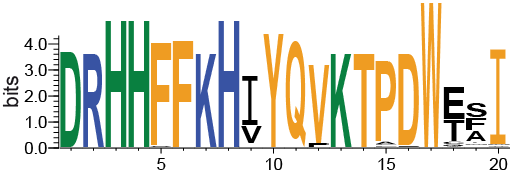 | 57 | Lactate racemases (WP_011100883.1 and WP_013298138.1 (LarA)) |
| Group 2 | 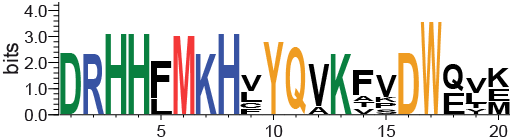 | 7 | Short-chain aliphatic α-hydroxyacid racemases (WP_013566321.1 (Sar)) |
| Group 3 | 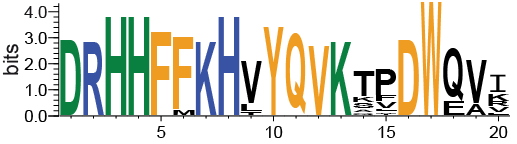 | 14 | Putative lactate racemases |
| Group 4 | 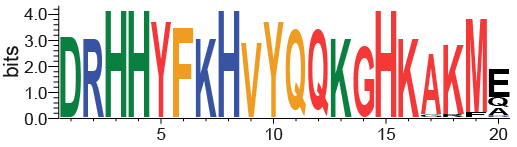 | 19 | Putative malate racemases |
| Group 5 | 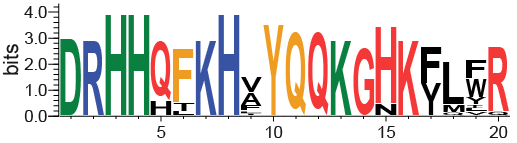 | 15 | Malate racemases (WP_015945206.1 (Mar1) |
| Group 6 | 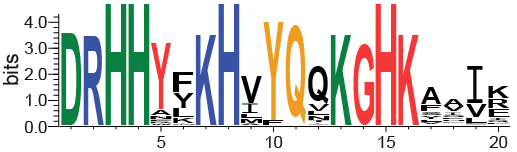 | 41 | Malate racemases (WP_013298851.1 (Mar2)) |
| Group 7a | 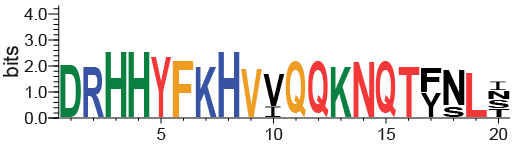 | 4 | α-Hydroxyglutarate racemases (WP_013008834.1 (Hgr)) |
| Group 7b | 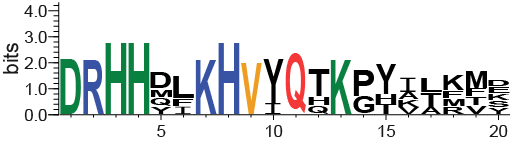 | 4 | Unknown function |
| Group 8 | 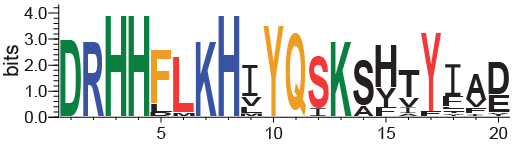 | 15 | Unknown function |
| Group 9 | 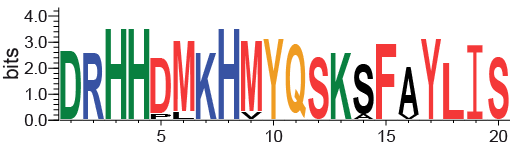 | 10 | Unknown function |
| Group 10 | 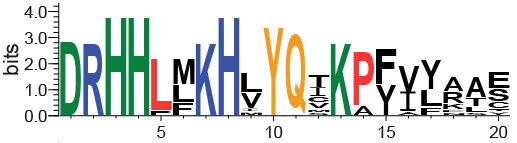 | 13 | Phenyllactate racemases (WP_014015216.1 (Plr)) |
| Group 11 | 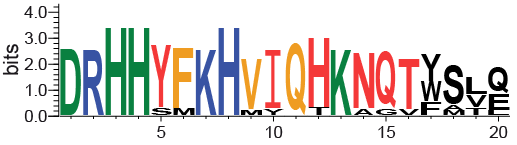 | 8 | Putative α-Hydroxyglutarate racemases |
| Group 14 | 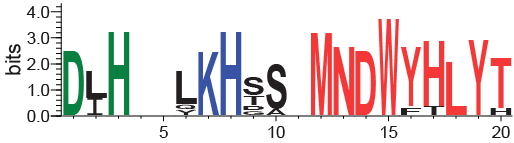 | 8 | Unknown function |
| Group 15 | 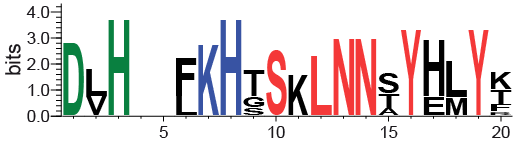 | 12 | Unknown function |
| Group 17 | 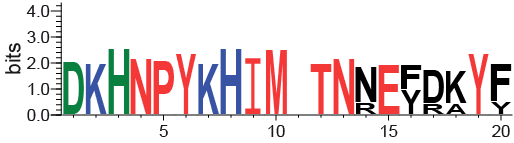 | 5 | Unknown function |
| Group 18 | 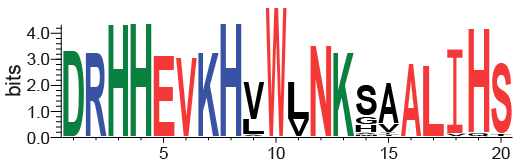 | 25 | Putative hexonate epimerase |
| Group 19 | 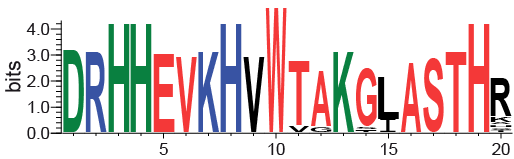 | 21 | Gluconate 2-epimerases (WP_004567503.1 (GntE1)) |
| Group 20 | 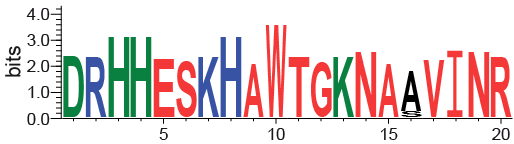 | 7 | Gluconate 2-epimerases (WP_010865122.1 (GntE2)) |
| Group 21 | 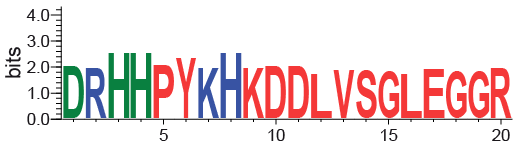 | 6 | Unknown function |
| Group 22 | 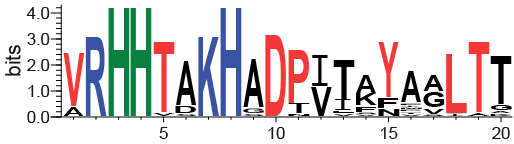 | 20 | Unknown function |
| Group 23 | 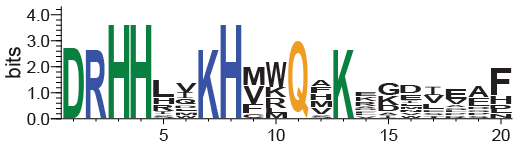 | 14 | Unknown function |
| Group 24 | 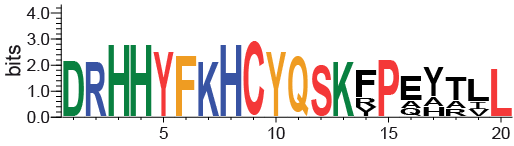 | 5 | Unknown function |
| Group 25 | 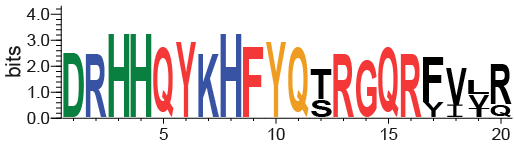 | 8 | Unknown function |

^a^The colors of the residues for each logo indicate those involved in catalytic activity (green), NPN binding (blue), probably involved in substrate recognition (yellow), conserved residues differing from LarA (red), and not conserved within the cluster (black). The logos were generated using Weblogo (3).

^b^Number of representative LarAHs

**Table S4.** Strains, plasmids, and primers.

| Strain, plasmid, or primer | Characteristic(s) or sequence |  | Source or reference |
| --- | --- | --- | --- |
| Strain |  |  |  |
| *Lc. Lactis* |  |  |  |
| NZ3900 | MG1363 derivative |  | (4) |
| *E. coli* |  |  |  |
| DH10B | F^-^ *endA*1 *recA*1 *galE*15 *galK*16 *nupG rpsL* Δ*lacX*74 Φ80*lacZ*ΔM15 *araD*139 Δ(*ara*,*leu*)7697 *mcrA* Δ(*mrr-hsdRMS*-*mcrBC*) λ^-^ | | Invitrogen |
| ArticExpress | Contains *Cpn60* and *Cpn10* from *Oleispira antarctica* | | Agilent |
|  |  |  |  |
| Plasmid |  |  |  |
| pNZ8048 | Cm^r^, P*_nisA_* |  | (5) |
| pGIR026 | Em^r^ Ap^r^; pGIZ660 with DNA encoding the StrepII-tag translationally fused at the 3ʹ-end of the *larB* ORF | LarB purification | (6) |
| pGIR031 | Cm^r^; pNZ8048 with DNA encoding the StrepII-tag sequence translationally fused to *larC* | LarC purification | (6) |
| pGIR076 | Amp^r^; pBADHisA with DNA encoding *larE* translationally fused to the StrepII-tag sequence | LarE  purification | (7) |
| pGIR210 | Derived from pGIR112 (17) without LarA, generated by recircularization of PCR product with pGIR210_A and B | pGIR21X plamsids | This study |
| pGIR211 | Cm^r^; pGIR210 with DNA encoding LarAH6 | LarAH purification in *L. lactis* | This study |
| pGIR212 | Cm^r^; pGIR210 with DNA encoding LarAH7 |  | This study |
| pGIR212 | Cm^r^; pGIR210 with DNA encoding GntE |  | This study |
| pGIR311 | Amp^r^; pBADHisA with DNA encoding LarAH3 | LarAH purification in *E. coli* | This study |
| pGIR312 | Amp^r^; pBADHisA with DNA encoding LarAH4 |  | This study |
| pGIR313 | Amp^r^; pBADHisA with DNA encoding LarAH5 |  | This study |
| pGIR314 | Amp^r^; pBADHisA with DNA encoding LarAH8 |  | This study |
| pGIR315 | Amp^r^; pBADHisA with DNA encoding LarAH10 |  | This study |
| pGIR316 | Amp^r^; pBADHisA with DNA encoding LarAH12 |  | This study |
| pGIR317 | Amp^r^; pBADHisA with DNA encoding LarAH14 |  | This study |
| pGIR318 | Amp^r^; pBADHisA with DNA encoding LarAH19 |  | This study |
| pGIR320 | Amp^r^; pBADHisA with DNA encoding LarAH23 |  | This study |
| pGIR390 | Amp^r^; pBADHisA with DNA encoding TnaA |  | This study |
|  |  |  |  |
| Primers |  |  |  |
| pGIR210_A | 3’-TTTGGTACCTGCTAGCTGGAGTCATCCACAATTTGAG  AAGTAG-5’ | pGIR210 | This study |
| pGIR210_B | 3’-CGGACATGTTGAGTGCCTCCTTATAATTTATTTTG-5’ | pGIR210 | This study |
| pGIR212_A | 3’-CCACCATGGCGACGGTGTATCTGGAAGGAGATCC-5’ | pGIR212 | This study |
| pGIR212_B | 3’-CCCGCTAGCTCCTCTCCTCTCCTTCAGATCGTAGAG-5’ | pGIR212 | This study |
| pGIR312_A | 3’-TTTACATGTCTCAAGTGAAAGTGCCTTATGGAAAAG-5’ | pGIR312 | This study |
| pGIR312_B | 3’-TTTGCTAGCTGGTTGGCTCACGACCTC-5’ | pGIR312 | This study |
| pGIR390_A | 3’-TTTCCATGGCAAGTTGGTCTCATCCACAATTCGAG  AAAGCTAGCGAAAACTTTAAACATCTCCCTGAAC-5’ | pGIR390 | This study |
| pGIR390_B | 3’-CCCAAGCTTAAACTTCTTTAAGTTTTGCGGTG-5’ | pGIR390 | This study |
|  |  |  |  |

**Dataset S1 (separate file).** Multiple alignment of the selected LarAH.

**Dataset S2 (separate file).** Multiple alignment of all 354 LarAH for the residues corresponding to the residues D72, R75, H108, H174, F175, F176, K184, H200, I220, Y294, Q295, V297, K298, T353, P355, D356, W358, T359, A360, and I362 of LarA.

**References**

1 Huson, D. H. & Scornavacca, C. Dendroscope 3: an interactive tool for rooted phylogenetic trees and networks. *Syst. Biol.* **61**, 1061-1067 (2012).

2 Pettersen, E. F. *et al.* UCSF Chimera--a visualization system for exploratory research and analysis. *Journal of computational chemistry* **25**, 1605-1612 (2004).

3 Crooks, G. E., Hon, G., Chandonia, J. M. & Brenner, S. E. WebLogo: a sequence logo generator. *Genome Res.* **14**, 1188-1190 (2004).

4 de Ruyter, P. G., Kuipers, O. P. & de Vos, W. M. Controlled gene expression systems for *Lactococcus lactis* with the food-grade inducer nisin. *Appl. Environ. Microbiol.* **62**, 3662-3667 (1996).

5 Kuipers, O. P., de Ruyter, P. G. G. A., Kleerebezem, M. & de Vos, W. M. Quorum sensing-controlled gene expression in lactic acid bacteria. *J. Biotechnol.* **64**, 15-21 (1998).

6 Desguin, B. *et al.* Lactate racemase is a nickel-dependent enzyme activated by a widespread maturation system. *Nat. Commun.* **5**, 3615 (2014).

7 Fellner, M., Desguin, B., Hausinger, R. P. & Hu, J. Structural insights into the catalytic mechanism of a sacrificial sulfur insertase of the N-type ATP pyrophosphatase family, LarE. *Proc. Natl. Acad. Sci. U. S. A.* **114**, 9074-9079 (2017).
